# Integrating network pharmacology and animal experimental validation to investigate the action mechanism of oleanolic acid in obesity

**DOI:** 10.1186/s12967-023-04840-x

**Published:** 2024-01-21

**Authors:** Tianfeng Liu, Jiliang Wang, Ying Tong, Lele Wu, Ying Xie, Ping He, Shujue Lin, Xuguang Hu

**Affiliations:** School of Chinese Materia Medica, Guangdong Pharmaceutical University, Waihuan East Road, Guangzhou, 510006 Guangdong China

**Keywords:** Oleanolic acid, Obesity, Network pharmacology, Molecular dynamics, PPARG/PPAR$\gamma$, PPAR signaling pathway, Lipid accumulation

## Abstract

**Background:**

Obesity, a condition associated with the development of widespread cardiovascular disease, metabolic disorders, and other health complications, has emerged as a significant global health issue. Oleanolic acid (OA), a pentacyclic triterpenoid compound that is widely distributed in various natural plants, has demonstrated potential anti-inflammatory and anti-atherosclerotic properties. However, the mechanism by which OA fights obesity has not been well studied.

**Method:**

Network pharmacology was utilized to search for potential targets and pathways of OA against obesity. Molecular docking and molecular dynamics simulations were utilized to validate the interaction of OA with core targets, and an animal model of obesity induced by high-fat eating was then employed to confirm the most central of these targets.

**Results:**

The network pharmacology study thoroughly examined 42 important OA targets for the treatment of obesity. The key biological processes (BP), cellular components (CC), and molecular functions (MF) of OA for anti-obesity were identified using GO enrichment analysis, including intracellular receptor signaling, intracellular steroid hormone receptor signaling, chromatin, nucleoplasm, receptor complex, endoplasmic reticulum membrane, and RNA polymerase II transcription Factor Activity. The KEGG/DAVID database enrichment study found that metabolic pathways, PPAR signaling pathways, cancer pathways/PPAR signaling pathways, insulin resistance, and ovarian steroidogenesis all play essential roles in the treatment of obesity and OA. The protein-protein interaction (PPI) network was used to screen nine main targets: PPARG, PPARA, MAPK3, NR3C1, PTGS2, CYP19A1, CNR1, HSD11B1, and AGTR1. Using molecular docking technology, the possible binding mechanism and degree of binding between OA and each important target were validated, demonstrating that OA has a good binding potential with each target. The molecular dynamics simulation’s Root Mean Square Deviation (RMSD), and Radius of Gyration (Rg) further demonstrated that OA has strong binding stability with each target. Additional animal studies confirmed the significance of the core target PPARG and the core pathway PPAR signaling pathway in OA anti-obesity.

**Conclusion:**

Overall, our study utilized a multifaceted approach to investigate the value and mechanisms of OA in treating obesity, thereby providing a novel foundation for the identification and development of natural drug treatments.

**Supplementary Information:**

The online version contains supplementary material available at 10.1186/s12967-023-04840-x.

## Introduction

Obesity, as a chronic and sophisticated modern global epidemic [1], has been found to reduce life expectancy and increase the risk of numerous illnesses [2]. Obesity prevalence in the United States increased from 30.5% to 42.4% between 1999 and 2018. Meanwhile, according to the most recent WHO report on obesity in Europe, 59% of adults and nearly one-third of children in Europe are overweight or obese. This imposes a substantial burden on millions of households [3–5]. Early intervention against obesity is an appropriate approach to prevent complex multimorbidity [6]. However, with certain anti-obesity medications producing mediocre outcomes and some reports of specific side effects, treating obesity is still difficult [7]. Particularly, many of these medications exhibit a high incidence of adverse effects related to cardiovascular diseases, leading to their withdrawal from the market [8], which further underscores the challenge of effectively managing obesity. Against this backdrop, there is growing interest in exploring the potential of Chinese herbal medicines derived from natural resources for the management of obesity and obesity-related diseases [9–11].

Oleanolic acid (OA), a pentacyclic triterpenoid, is an active ingredient in herbs such as Fructus Ligustri Lucidi and Folium Camellia Sinensis, as well as in numerous common foods including olive leaves, Apple, Grape, Ginger, and Mango [12]. In China, OA has been utilized as a hepatoprotective medication [13], and there is clinical proof of its effectiveness in treating hyperlipidemia [14]. Furthermore, multiple clinical studies have demonstrated the potential of various OA derivatives to prevent or treat a range of diseases, including cancer, diabetes, and viral infections [13, 15, 16]. Previous research has identified OA as possessing diverse pharmacological properties, such as antiviral, antibacterial, anticancer, anti-inflammatory, antioxidant, hepatoprotective, and gastroprotective effects [17–19]. OA has been shown to improve aberrant alterations in lipid parameters, reduce hepatic microvesicular steatosis, increase leptin content, significantly reduce visceral fat, improve glucose tolerance, and elevate insulin levels [20, 21]. It also reduced systemic inflammation, promoted hepatic lipogenesis, and enhanced the taste perception of dietary fat in mice fed the HFD [21]. Meanwhile, OA derivatives, such as Nano-OA, exhibit similar lipid-lowering effects [22]. Although the hypolipidemic effect of OA has been confirmed [23, 24], further research is required to elucidate the mechanisms underlying its potential role in combating obesity.

Network pharmacology is an important approach for investigating the biological effects of small molecules derived from various natural resources by constructing biomedical interaction networks to assess drug molecular mechanisms [25, 26]. Particularly, network pharmacology is an efficient tool for identifying active ingredients and potential targets of Chinese medicine [27]. Molecular docking and molecular dynamics techniques can provide an in-depth account of intermolecular interactions can graphically explain the mechanism of interactions, and have equally important applications in drug development [28]. Consequently, combining molecular simulation-assisted validation with network pharmacological analysis screening is a useful approach to examine the anti-obesity mechanism of OA.

To shed light on the underlying mechanism of OA’s anti-obesity effect, the present study employs a combination of network pharmacology, molecular docking, and molecular dynamics simulation techniques to screen and analyze key targets and pathways. Moreover, in vivo experiments are conducted to validate the principal targets and pathways. As far as we know, this represents the first systematic investigation into the anti-obesity effect and mechanism of OA. The study findings will not only serve as an experimental foundation for utilizing OA as an anti-obesity agent but also offer a theoretical basis for the application of OA-derived drugs and food, such as olive oil, in the management and prevention of obesity.

## Results

### Network pharmacology analysis

**Fig. 1:**
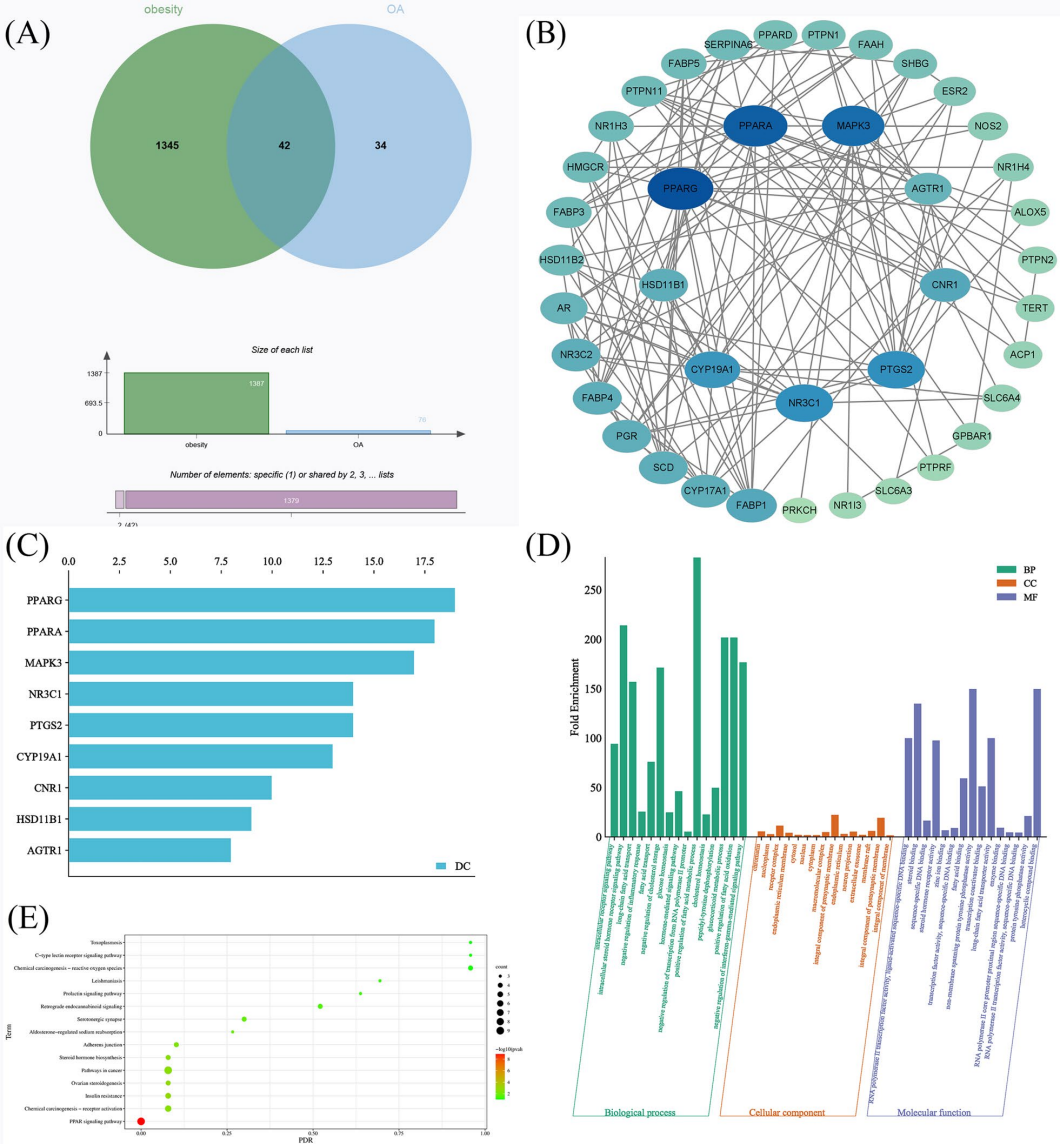
Analysis of the targets associated with OA and obesity. **A** Venn diagram of OA and obesity-associated targets. Includes 78 OA-related targets (left), 4556 obesity-related targets (right), and 42 OA obesity-related targets (center). **B** OA anti-obesity PPI network. A larger area indicates larger nodes, a Blue color indicates higher association, lighter color less association, and the core target is the target in the inner circle. **C** Degree values of core anti-obesity targets in OA. **D** GO enrichment analysis. fold enrichment (y-axis), term (x-axis); green, orange, and purple represent the 15 core results for BP, CC, and MF, respectively. **E** KEGG pathway enrichment analysis (DAVID). Pathways (Y-axis), FDR (X-axis), and P-values (color change). Bubble size indicates the number of genes enriched in the pathway

To investigate the potential molecular targets of OA for the management of obesity, a network pharmacology analysis was conducted(Figure 1). Initially, 78 genes relevant to OA (CC1(CCC2(CCC3(C(=CCC4C3(CCC5C4(CCC(C5(C)C)O)C)C)C2C1)C)C(=O)O)C) were identified utilizing the SwissTargetPrediction database, while 1,387 genes associated with obesity were sourced from the GeneCards database. Subsequently, a Venn diagram was constructed employing the microbiology letter platform, and 42 related targets were identified (Fig. 1A). To visually assess the impact of OA on obesity, we constructed a (PPI network consisting of 42 nodes and 148 edges, employing the identified intersecting genes (Fig. 1B). The primary targets of OA involved in its anti-obesity effect are illustrated in Fig. 1C, which comprises nine proteins. Notably, PPARG exhibited the highest number of connections, being linked to 19 additional proteins. Based on their associations, PPARA, MAPK3, NR3C1, PTGS2, CYP19A1, CNR1, HSD11B1, and AGTR1 were linked to 18, 17, 14, 13, 10, 9, and 8 additional proteins, respectively.

To provide a comprehensive and systematic illustration of the potential mechanisms of action of OA in treating obesity, we conducted a GO enrichment analysis of its therapeutic targets at different levels using the DAVID database. The analysis covered BP, CC, and MF. We identified 149 statistically significant GO terms, comprising 93 BP, 12 CC, and 44 MF terms. Bar charts were used to display the top 10 enrichment terms of BP, CC, and MF with the highest gene counts (Figure 1D). Our results showed that the targets of OA in treating obesity were predominantly enriched in biological processes such as intracellular receptor signaling, intracellular steroid hormone receptor signaling, long-chain fatty acid transport, negative regulation of the inflammatory response, and other biological responses. They were also enriched in cellular components such as chromatin, nucleoplasm, receptor complex, endoplasmic reticulum membrane, and other cellular components. Moreover, OA targets were found to exhibit molecular functions such as RNA polymerase II transcription factor activity, ligand-activated sequence-specific DNA binding, steroid binding, sequence-specific DNA binding, and other molecular functions.

**Fig. 2:**
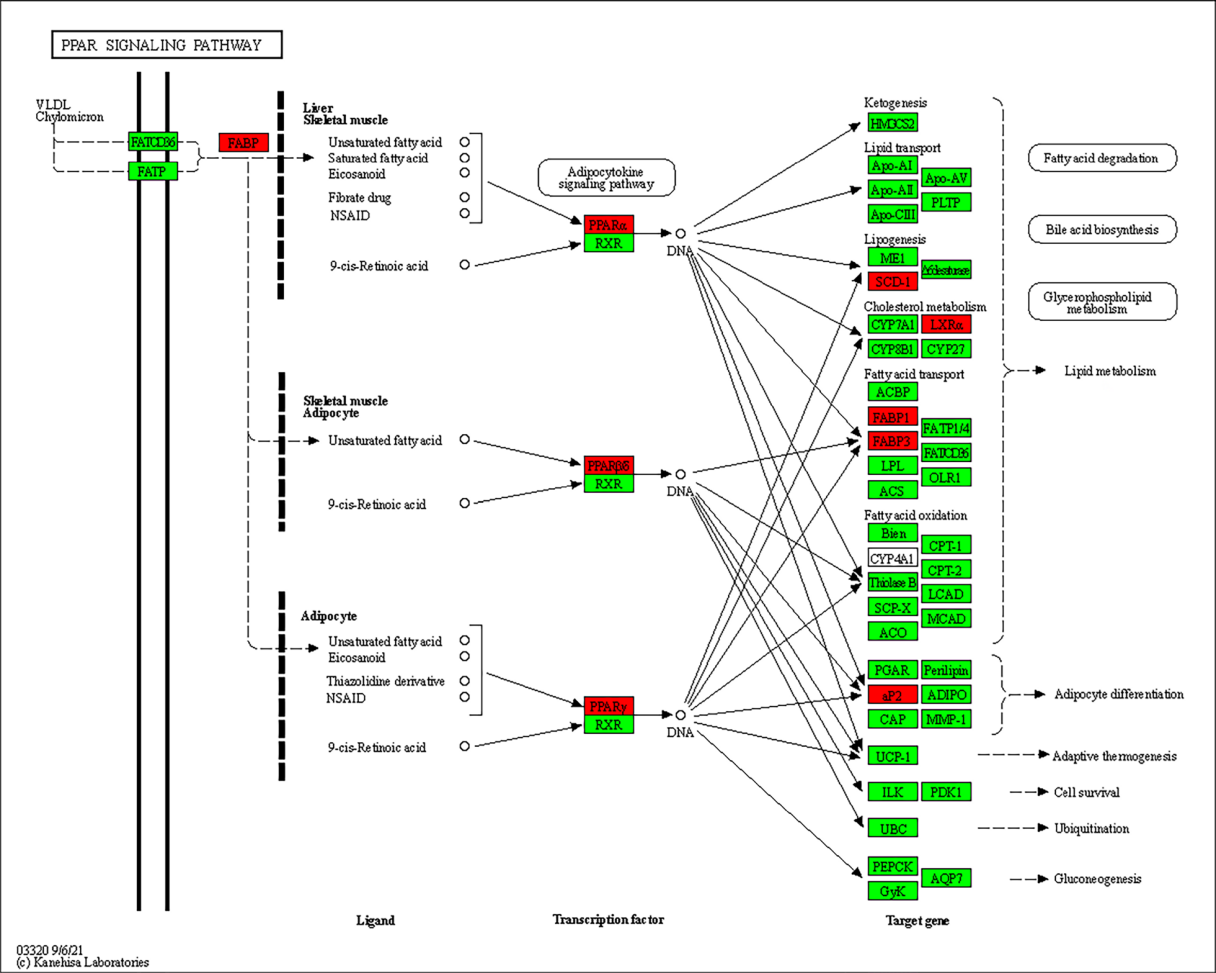
Diagrams of the PPAR signaling pathway. Highlighting the target that is common to both OA and obesity, represented in red color

To systematically investigate the potential mechanisms underlying the anti-obesity effects of OA, we utilized KEGG pathways to predict the signaling pathways of OA’s potential anti-obesity targets. The results revealed 172 statistically significant related pathways. The top ten pathways with the highest number of linked genes were presented in a bar graph in Table 1. Among them, the metabolic pathway, the PPAR signaling pathway, and the cancer pathway were the top three pathways. In addition, we performed KEGG enrichment analysis of OA anti-obesity targets using the DAVID database, which resulted in 16 relevant pathways (Figure 1E). The top three potential pathways were the PPAR signaling pathway, Insulin resistance, and ovarian steroidogenesis. Our investigation into the discrepancies in the outcomes of the two database studies may be connected to the database’s update. We focused on the PPAR signaling pathway based on the results of the enrichment of core targets and pathways, and the schematic diagram of OA in the treatment of obesity by targeting the PPAR signaling pathway is shown in Fig. 2. These findings imply that OA can exhibit anti-obesity benefits via a variety of routes, among which the PPAR signaling pathway plays a key role in OA’s anti-obesity process.

**Table 1:** The main pathway of OA against obesity (KEGG)

KEGG ID	Signaling pathway	Count
hsa01100	Metabolic pathways - Homo sapiens human	10
hsa03320	PPAR signaling pathway - Homo sapiens human	9
hsa05200	Pathways in cancer - Homo sapiens human	9
hsa05207	Chemical carcinogenesis - receptor activation - Homo sapiens human	6
hsa04931	Insulin resistance - Homo sapiens human	5
hsa04520	Adherens junction - Homo sapiens humann	4
hsa04723	Retrograde endocannabinoid signaling - Homo sapiens human	4
hsa00140	Steroid hormone biosynthesis - Homo sapiens human	4
hsa05022	Pathways of neurodegeneration - multiple diseases - Homo sapiens human	4
hsa05208	Chemical carcinogenesis - reactive oxygen species - Homo sapiens human	4

### Molecular docking

To investigate the mechanism of OA against obesity and to study the potential binding mode and degree of binding to key targets, we conducted molecular docking of OA and obesity-related core targets. The core anti-obesity targets of OA were selected for molecular docking, and all macromolecular structures were re-docked before formal docking. The RMSD values for all of them were less than 2A, indicating good precision and a good binding pattern [42]. It is widely accepted that a lower binding energy indicates a more stable binding of ligand and receptor, suggesting that the chemical is more likely to interact with the protein. The results in Table 2 demonstrate that all 9 target proteins can spontaneously bind to OA, and all of them have binding energies that are less than -20 kJ/mol, indicating potent binding ability. This suggests that PPARG, PPARA, and other targets should be the primary direct targets for OA to exert its anti-obesity effects.

**Table 2:** Results of the re-docking of core target structures

Targets	RMSD
PPARG	0.018
PPARA	0.038
MAPK3	0.031
NR3C1	0.006
PTGS2	0.037
CYP19A1	0.532
CNR1	0.027
HSD11B1	0.019
AGTR1	0.028

**Fig. 3:**
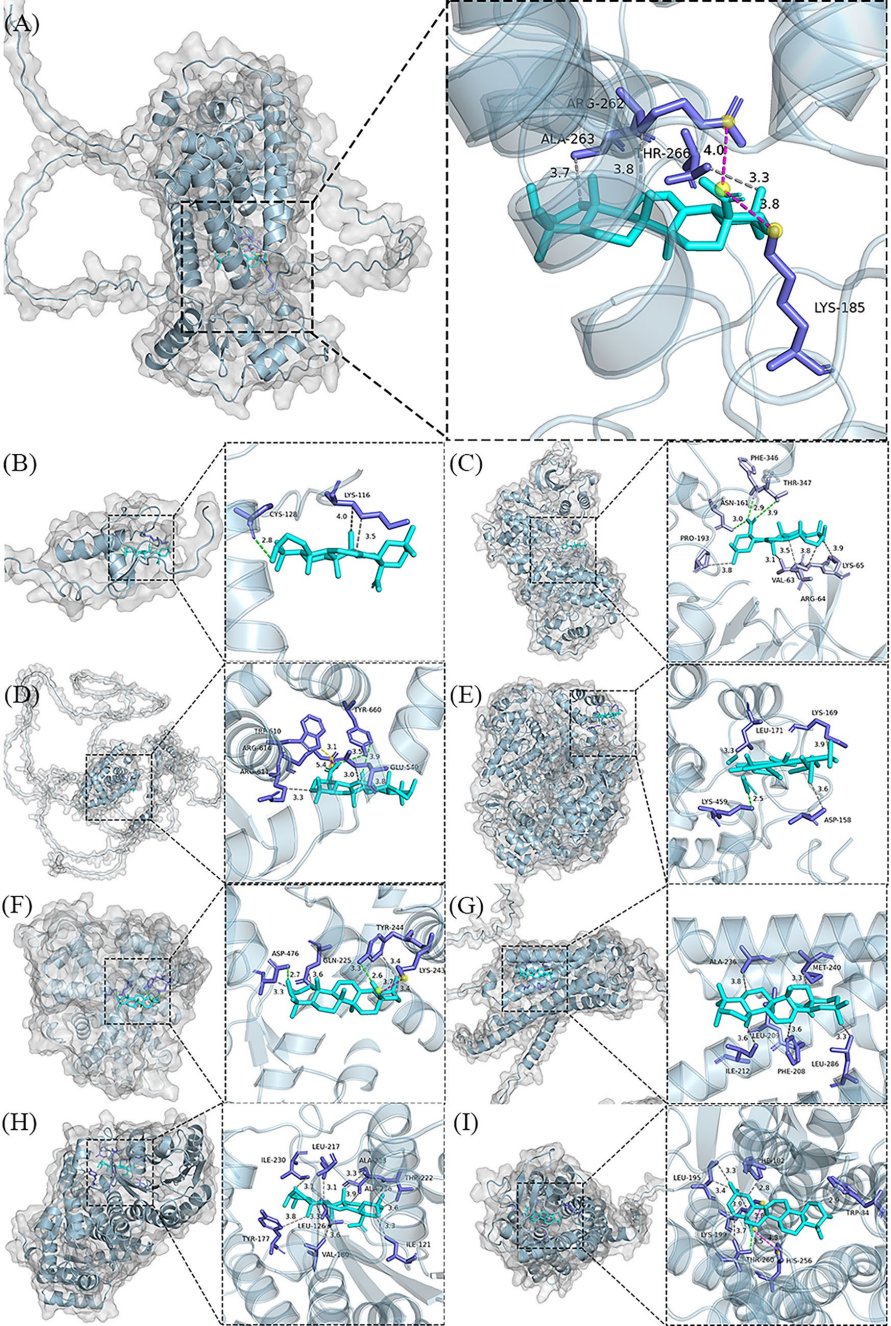
Molecular docking models of OA with possible core anti-obesity targets. **A** PPARG, **B** PPARA, **C** MAPK3, **D** NR3C1, **E** PTGS2, **F** CYP19A1, **G** CNR1, **H** HSD11B1, and **I** AGTR1

Molecular docking results indicate that OA interacts with key target proteins at the atomic level through non-covalent interactions, such as hydrophobic interactions, hydrogen bonds, and salt bridges. As shown in Fig. 3 and Table 3, OA can bind to HSD11B1, and CNR1 via hydrophobic interactions; connect to MAPK3, PPARA, and PTGS2 via hydrophobic interactions and hydrogen bonds; and is predicted to dock in the binding pocket of CYP19A1, AGTR1, NR3C1, and PPARG via hydrophobic interactions, hydrogen bonds, and salt-bridge interactions. Based on the docking studies, we effectively predicted the binding of OA to target proteins.

**Table 3:** Docking results of OA with core target molecules

Targets	Binding Energy (kJ/mol)	Hydrophobic Interactions	Hydrogen Bonds	Salt Bridges
PPARG	− 24.85	ARG 262, ALA 263, THR 266	–	LYS 185, ARG 262
PPARA	− 26.15	LYS 116, LYS 116	− CYS 128	–
MAPK3	− 24.18	VAL 63, ARG 64, ARG 64, LYS 65, PRO 193	ASN 161, PHE 346, THR 347	–
NR3C1	− 24.64	GLU 540, GLU 540, ARG 611, TYR 660	TRP 610, TYR 660	–
PTGS2	− 26.15	PHE 205, VAL 344, TYR 348	TYR 385, GLY 526, GLY 533, LEU 534	–
CYP19A1	− 29.20	GLN 225, LYS 243, TYR 244, TYR 244, ASP 476	TYR 244, ASP 476	LYS 243
CNR1	− 22.55	PHE 208, LEU 209, ILE 212, ALA 236, MET 240, LEU 286	–	–
HSD11B1	− 34.14	ILE 121, LEU 126, TYR 177, VAL 180, LEU 217, THR 222, ALA 223, ALA 226, ILE 230	–	–
AGTR1	− 31.59	TRP 84, PHE 182, LEU 195, LEU 195, LYS 199, THR 260	THR 260	LYS 199, HIS 256

In each model, the blue bar structures represent the OA molecules, while the purple structures represent the binding sites of residues in the target proteins. The interactions were visualized through dashed lines, where gray dashed lines represent hydrophobic interactions, green dashed lines represent hydrogen bonds, and magenta dashed lines represent salt bridges. Additionally, the labels in the models show the residues and the distance of the interactions between OA and the respective targets.

### Molecular dynamics simulation

Molecular dynamics simulations confirmed OA’s stability to attach to the core target (Additional file 1: Fig. S1). The structural variation at a given instant relative to the original conformation is referred to as the RMSD. The lower the score, the more stable OA’s binding conformation to the target site. The results demonstrate that the composite structures of OA with each core target are relatively stable, fluctuating mostly about 2 nm and beginning to remain stable around 15 ns, indicating that OA can be stably bound to each core target. Rg is the mass-weighted average radius of the system during the simulation and is often used to determine tightness and folding stability during protein-ligand binding. Additional file 1: Fig. S1B demonstrates that the values of OA complexed with each target are limited to 3.5 nm throughout the simulation, indicating that the complexes are compact. The complexes of PPARG, PPARA, and PTGS2 are particularly subdued, indicating that they are more stable. Taken together, our molecular dynamics results suggest that OA has binding stability to core targets, especially PPARG, PPARA, and PTGS2, which have very high potential in OA anti-obesity.

### Animal experiment

#### OA prevents weight gain in obese mice

**Fig. 4:**
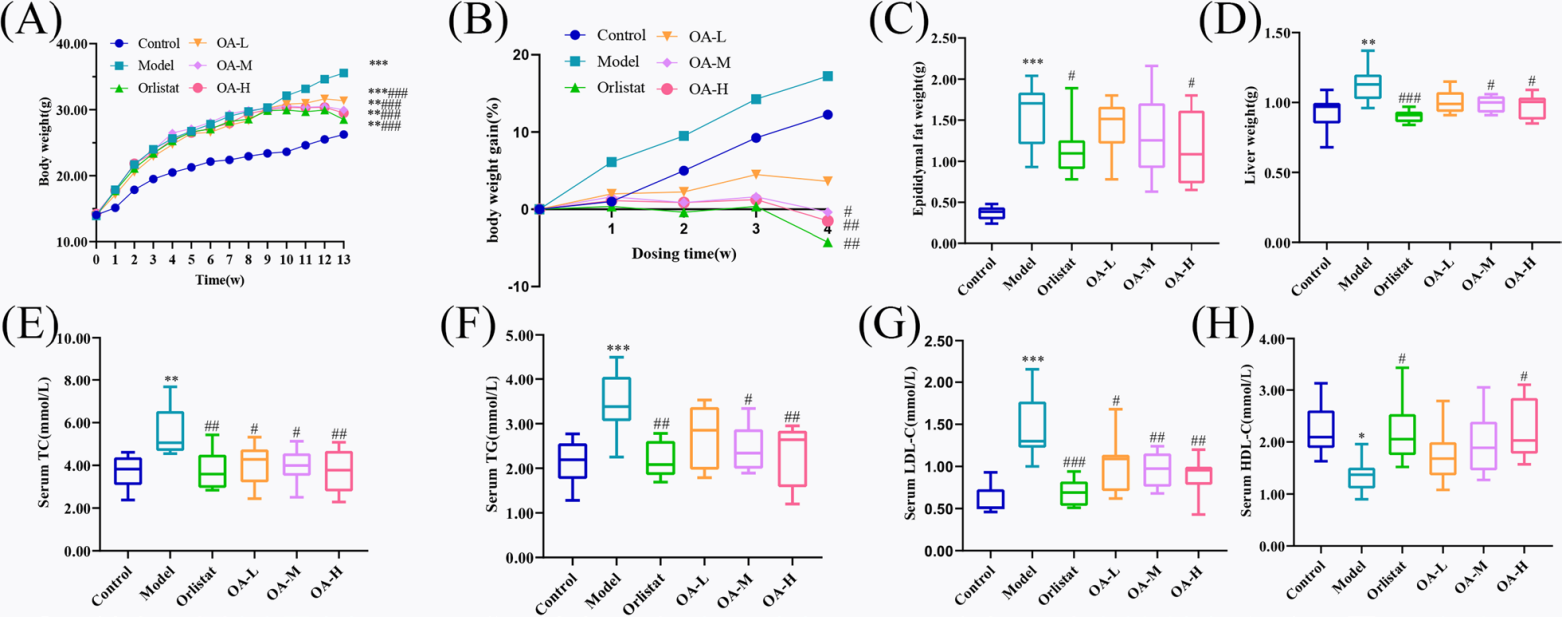
Effect of OA on lipid metabolism in high-fat-fed mice. **A** Changes in body weight during feeding, **B** Body weight gain in each group after administration, **C** Liver weight, **D** epididymal fat weight. **E** TC, **F** TG, **G** LDL-C, **H** HDL-C.compared to control, $${}^{*}{P }{< 0.05}$$, $${}^{**}{P }{< 0.01}$$, $${}^{***}{P }{< 0.001}$$; compared to model, $${}^{\#}{P }{< 0.05}$$, $${}^{\#\#}{P }{< 0.01}$$, $${}^{\#\#\#}\,{P }{< 0.001}$$

The continuous feeding of HFD to mice resulted in a rapid increase in body weight, with a significant difference observed between HFD and control mice early on (*P*$< 0.001$). Changes in body weight in each group were evident after administration commenced at week 10. After 4 weeks of administration, mice in the orlistat, OA-L, OA-M, and OA-H groups showed a significant decrease in body weight compared to the model group, and the difference was statistically significant (*P*<0.001) (Fig. 4A). Additionally, the percentage change in body weight after dosing decreased in each dosing group, which was significantly lower than the change in body weight in the model group, and the percentage change in body weight after dosing was negative in the orlistat, OA-M, and OA-H groups, which was a significant difference (Fig. 4B). These findings suggest that OA effectively retards weight gain in HFD-induced obese mice and even induces weight loss.

#### OA regulates disorders of glycolipid metabolism

The weight of the mice’s liver and epididymal fat increased as a result of the HFD diet, and the animals in the model group had considerably higher indices than the mice in the control group. Upon treatment, the orlistat, OA-L, OA-M, and OA-H groups displayed reduced liver weight and epididymal fat compared to the model group (Fig. 4C, D). The OA-H and orlistat groups were found to be the most effective with statistically significant differences in their effects. These results suggest that OA reduces fat accumulation in obese mice.

Prolonged exposure to HFD led to a significant increase in FBG levels in the model mice (*P*$< 0.001$), which was notably ameliorated by treatment with orlistat (*P*$< 0.001$), as well as with low (*P*$< 0.01$), medium (*P*$< 0.001$), and high doses (*P*$< 0.001$) of OA. Moreover, the HFD diet elevated the lipid levels in the mice, causing a significant increase in TG (*P*$< 0.001$), TC (*P*$< 0.01$), and LDL-C (*P*$< 0.001$), and a significant decrease in HDL-C (*P*$< 0.05$) in the model group. After treatment, orlistat (TG: *P*$< 0.01$, TC: *P*$< 0.01$, LDL-C: *P*$< 0.001$, HDL-C: *P*$< 0.05$), low (TC: *P*$< 0.05$, LDL-C: *P*$< 0.05$), medium (TG: *P*$< 0.05$, TC: *P*$< 0.05$, LDL-C: *P*$< 0.01$), and high (TG: *P*$< 0.01$, TC: *P*$< 0.01$, LDL-C: *P*$< 0.01$, HDL-C: *P*$< 0.05$) doses exhibited remarkable effects on lipid regulation, as evidenced by lower levels of TG, TC, LDL-C, and increased levels of HDL-C (Figure 4E–H). These results suggest that OA has a favorable impact on regulating glucolipid metabolism, with a dose-dependent effect.

#### OA improves tissue pathological changes in HFD-fed mice

**Fig. 5:**
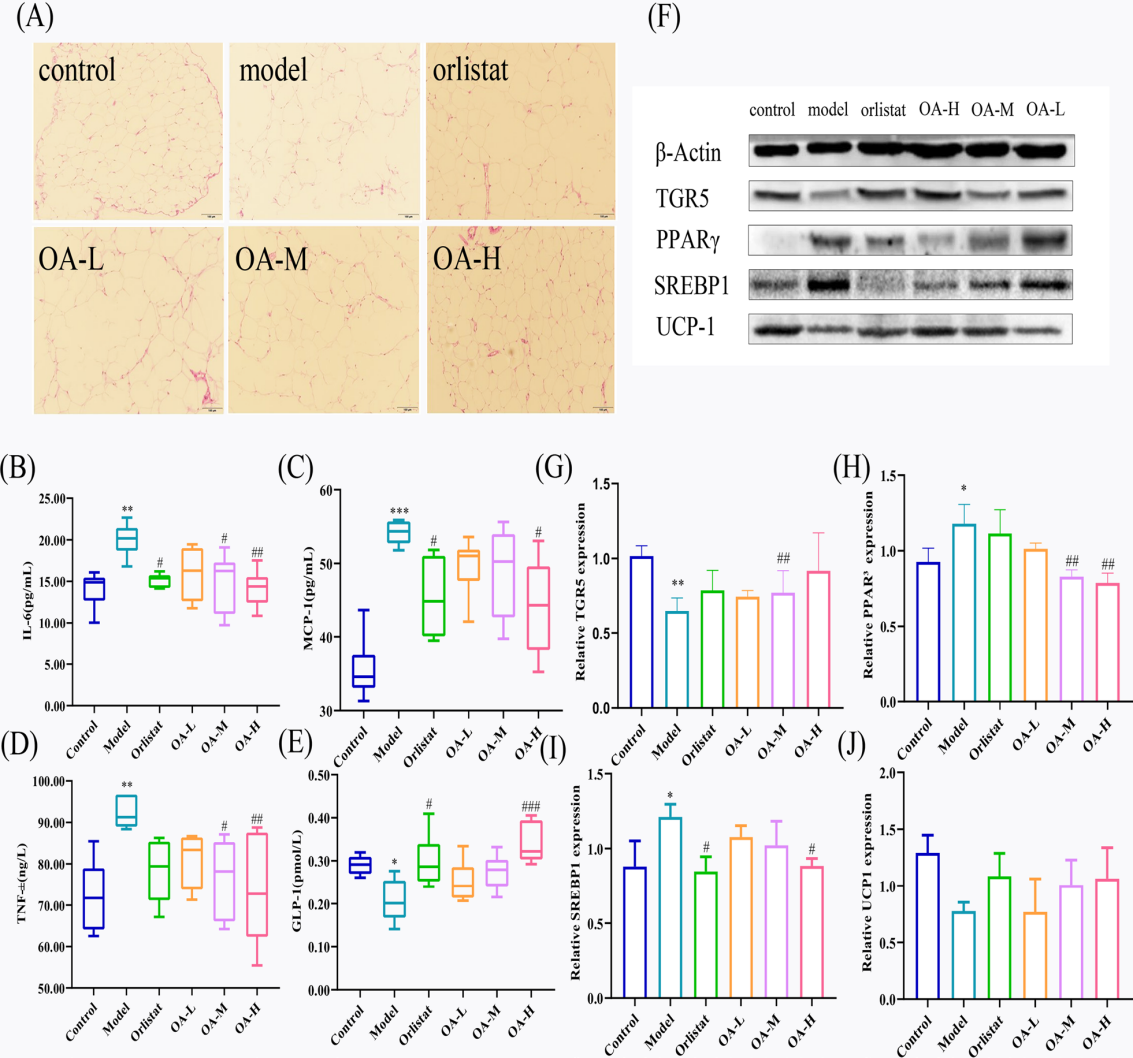
Effect of OA on lipid metabolism in high-fat-fed mice. **A** The effect of OA on fat tissue damage in high-fat fed mice. **B** IL-6, **C** MCP-1, **D** TNF-$$\alpha$$, **E** GLP-1, **F** Protein blot detection strip map, **G** TGR-5, **H** PPAR$$\gamma$$, **I** SREBP1, **J** UCP-1. compared to control, $${}^{*}{P }{< 0.05}$$, $${}^{**}{P }{< 0.01}$$, $${}^{***}{P }{< 0.001}$$; compared to model, $${}^{\#}{P }< 0.05$$, $${}^{\#\#}{P }{<} 0.01$$, $${}^{\#\#\#}{P }{<} 0.001$$

The increased and bloated adipocyte volume and many ruptured adipocyte cytoplasm are signs of adipose tissue injury in HFD-induced animals, as illustrated in Fig. 5A. Orlistat and OA-H showed a more noticeable effect. Administration with OA did, however, result in some alleviation of tissue damage. The ability of OA to reduce tissue damage and improve steatosis in obese mice fed a high-fat diet is highlighted by this.

#### OA alleviates HFD-induced inflammation response

The feeding of HFD led to a substantial elevation in the levels of serum cytokines, specifically IL-6 (*P*$< 0.01$), TNF-$\alpha$ (*P*$< 0.01$), and chemokine monocyte MCP-1 (*P*<0.001). Fortunately, medication therapy was successful in reducing the levels of these inflammatory markers, as illustrated in Fig. 5B–D. Of note, high doses of OA demonstrated anti-inflammatory potential comparable to that of orlistat. These results suggest that OA attenuates the inflammatory response in obese mice, which may contribute to its anti-obesity effects.

#### OA increases GLP-1 secretion by regulating TGR5

The data presented in Fig. 5E indicate that the HFD mice had significantly lower serum GLP-1 levels (*P*< 0.05). Notably, the orlistat (*P*< 0.05) and OA-H (*P*< 0.001) groups exhibited a significant increase in GLP-1 levels in all animals. Protein blotting tests revealed that HFD diet reduced TGR5 expression, with considerably lower data in the model group (*P*< 0.01). Moreover, orlistat and all doses of OA (OA-M: *P*< 0.01) resulted in decreased obesity-related protein inhibition and increased TGR5 expression (Fig. 5G). It should be noted that only TGR5 was detected due to experimental limitations, and further studies are necessary to validate this finding. Nevertheless, based on the experimental results, we speculate that OA enhances GLP-1 secretion through TGR5 upregulation, thereby exerting anti-obesity and anti-insulin resistance effects.

#### OA affects lipid synthesis via PPAR$\gamma$/SREBP1

To confirm the previously hypothesized targets and pathways of OA for anti-obesity effects, we conducted protein blotting to detect the expression of the key adipogenesis factors PPAR$\gamma$ and SREBP1. The impact of a high-fat diet resulted in an increase in the relative protein expression of both PPAR$\gamma$ (0.05) and SREBP1 (*P*< 0.05) in white adipose tissue samples of obese mice in the model group, which corresponded to an increase in body fat mass. Treatment with orlistat and OA differentially prevented the increase of both proteins. Among them, OA appeared to have a better effect in regulating PPAR$\gamma$, with both OA-M (*P*< 0.01) and OA-H (*P*< 0.01) significantly decreasing PPAR$\gamma$ expression (Fig. 5H). In the regulation of SREBP1, OA (*P*< 0.01) had a similar effect to orlistat (*P*< 0.01) (Fig. 5I). Combined with the data from the animal sera, it is reasonable to suggest that OA can control obesity by regulating PPAR$\gamma$/SREBP1 to control lipid synthesis.

#### OA affects energy consumption with TGR5/UCP-1

We also explored the effect of OA on energy consumption by studying the expression of TGR5/UCP-1 protein. The expression of TGR5 is described above. Compared with the control group, the UCP1 in the white adipose tissue of mice in the model group was significantly reduced (*P*< 0.01) (Fig. 5J). At the same time, by dosing, the expression of each treatment group was increased, although none of them were statistically significant. Based on the results of protein expression, we can hypothesize that one of the mechanisms of OA regulation of obesity is the enhancement of TGR5 / UCP-1 protein expression, hence accelerating energy expenditure. However, this hypothesis still needs to be validated by other research.

## Methods

### Network pharmacology analysis

#### Obtaining targets associated with OA and obesity

The PubChem database (https://pubchem.ncbi.nlm.nih.gov/) [29] was utilized to obtain the SMLLE name of “Oleanolic acid”, which was then imported into the SwissTargetPrediction database (http://swisstargetprediction.ch/) [30] to obtain OA’s corresponding targets (probability$>0$). Next, the GeneCards database (https://www.genecards.org/) [31] was searched for disease-related targets using the keyword “obesity”, followed by applying filters (Relevance score$>2$) to extract the final set of disease targets. The two lists of targets obtained were then analyzed using the bioinformatics platform (http://www.bioinformatics.com.cn/) to identify the targets that overlap between OA and obesity.

#### Constructing protein-protein interaction (PPI) network

The identification of therapeutic targets of OA against obesity was achieved by overlaying the targets of OA with those related to obesity. The therapeutic targets were then subjected to analysis using the STRING database (https://string-db.org/) [32] to construct a protein-protein interaction (PPI) network. The resulting PPI network was subsequently visualized with the aid of Cytoscape 3.9.1 software [33].

#### Screening core targets of OA anti-obesity

The CytoNCA plug-in [34] for Cytoscape was then used to analyze and evaluate the Betweenness (BC), Closeness (CC), Degree (DC), and Eigenvector (EC) values for each target. Targets that exhibited scores above the mean value for each metric were selected and intersected to obtain the essential targets of OA for treating obesity.

### Gene ontology (GO) enrichment analysis

The targets related to OA and obesity were subjected to GO functional enrichment analysis using the DAVID database (https://david.ncifcrf.gov/) [35] under the identifier restriction “OFFICIAL_GENE_SYMBOL” and species “homo sapiens”. The resulting data were used to generate statistical graphs for CC, MF, BP, and Kyoto Encyclopedia of Genes and Genomes (KEGG) using a bioinformatics platform (http://www.bioinformatics.com.cn/).

#### KEGG pathway enrichment analysis

The therapeutic targets of OA were converted from “gene name” to “UniProtKB” form using the UniProt database (https://www.uniprot.org) [36]. Next, the KEGG IDs were obtained, and the target’s pathway information was matched using KEGG Mapper (https://www.genome.jp/kegg/mapper/). The related gene colors in the pathway were then modified for subsequent analysis and prediction of the OA therapeutic pathway against obesity.

### Molecular docking analysis

To investigate the interaction between OA and potential anti-obesity targets, the core targets identified by network pharmacology analysis were used as docking receptors, and OA was utilized as ligands for molecular docking via AutoDock. Initially, the 3D structure of OA was obtained in “mol2” format from the China Traditional Medicine System Drug Database and Analysis Platform (TCMSP, https://old.tcmsp-e.com/tcmsp.php) [37], and then saved in “PDB” format using Pymol 2.5.7 (Portland, OR, US). The protein structure of the core targets in “PDB” format was downloaded from protein databases PDB (https://www.rcsb.org) [38] and the alphafold databases (https://alphafold.com) [39]. Next, the ligands of the target macromolecules were removed using Pymol software, and the molecules were pretreated using AutoDock tool 1.5.7 software, including hydrogenation, electron addition, and root addition. In the final step, AutoDock 4.2.6 software was employed for molecular semi-flexible docking, and the binding potential was assessed by an affinity score. The optimal docking model was then selected, and the noncovalent interactions of the protein-ligand complex at the atomic level were analyzed by the Protein-ligand Interaction Profiler (PLIP) web tool (https://plip-tool.biotec.tu-dresden.de/plip-web/plip/index) [40]. Finally, redocking was conducted to evaluate binding patterns before the formal molecular docking, followed by visual analysis using Pymol software.

### Molecular dynamics simulation

Molecular dynamics simulations of OA and core targets were carried out using GROMACS software (2022.5) to more precisely validate the predictions of network pharmacology and to minimize the error between the conformations obtained by docking proteins and small molecules and the realistic complexes. Software SPDBV4.10 was used to repair the protein structure. Pymol 2.5.7 was used to save the molecularly docked small molecule ligands in mol2 format. Sobpob 1.0 (dev3.1) was used to process the ligand and topology files. GROMACS software was then used to process the protein and topology files, add boxes, water, and ions, equilibrate the ions, minimize the energies, and Nvt preequilibrium, and finish the kinetic simulation procedure. Following the simulation, the Root Mean Square Deviation (RMSD) and Radius of Gyration (Rg) were evaluated by GROMACS to analyze the stability of the protein-ligand binding.

### In vivo assay

#### Chemicals and antibodies

OA (HPLC $\ge$ 98$\%$) was supplied from Yuanye (Shanghai, China) and dissolved by 2$\%$ Tween 80 phosphate-buffered saline (PBS pH:7.2-7.4). Orlistat capsules were purchased from Aili (Chongqing, China). The kits of TC, TG, HDL-C, LDL-C, ALT, AST, GSH, and MDA were obtained from Nanjing Jiancheng Biotechnology Co. (Nanjing, Jiangsu, China). The enzyme-linked immunosorbent assay (ELISA) kits of interleukin 6 (IL-6), tumor necrosis factor-$\alpha$(TNF-$\alpha$), Glucagon-Like Peptide 1 (GLP-1) and Monocyte Chemotactic Protein 1 (MCP-1) was supplied from Meimian (Yancheng, Jiangsu, China). The primary antibodies of SREBP-1, TGR5, UCP-1, and $\beta$-actin were purchased from Abclonal (Wuhan, Hubei, China). Meanwhile, Abcam (Cambridge, UK) provides the secondary antibodies and PPAR$\gamma$ antibody.

#### Experimental animals and feeding conditions

Fifty 4-week-old male C57BL/6J mice (15±1g) were purchased from Guangdong Medical Laboratory Animal Center (Guangzhou, Guangdong, China) and were kept in an SPF level environment with 12 hours of light and dark cycles, controlled room temperature of (25±2) $^\circ$C and relative humidity of (50±10)$\%$. The animal experiments conducted during this research were by the animal care protocols approved by Guangdong Pharmaceutical University’s Animal Protection and Use Ethics Committee.

#### Grouping and model establishment

The animal experimental protocol of this study was conducted by previously established procedures [41]. After five days of acclimation feeding, 50 male C57BL/6J mice were divided into six groups at random: control, model, orlistat (15.6mg/kg/d), oleanolic acid low dose (OA-L, 25mg/kg/d), oleanolic acid medium dose (OA-M, 50mg/kg/d), and oleanolic acid high dose (OA-H, 100mg/kg/d) groups. The experiment lasted 13 weeks in total. To establish the model, a high-fat diet was given for 9 weeks, followed by medication administered via gavage for 4 weeks. The control group and the other five groups were fed the following two diets respectively: (1) ND (normal diet, Wheat flour, soybean cake flour, corn flour, fish flour, bran, yeast Powder, bone meal, salt, cod liver oil, mineral additives, n = 10); (2) HFD (high-fat diet, 60% of energy from fat; HF60, n = 40). The high-fat diets were obtained from Dyets, Inc. The modelling was considered successful when the mean body weight of mice in the NFD group exceeded 20% of the ND.

#### Administration and handling

Starting at week 10, each group was administered saline, orlistat (15.6 mg/kg/d), or varying doses of OA (25 mg/kg/d, 50 mg/kg/d, and 100 mg/kg/d) via gavage for four weeks. Throughout the study period, the mice’s body weights were measured weekly. The mice were euthanized under anesthesia at the end of the experiment after a 12-hour fast with access to water. Blood was removed from the eyeball and left to stand at room temperature for an hour before being centrifuged for 10 minutes at 3500 rpm to separate it. The liver, epididymal fat, caecal contents, and brown adipose tissues of the mice were removed after dissection and either immediately frozen in liquid nitrogen or preserved with paraformaldehyde.

#### Calculation of weight gain and weight measurement of liver and epididymal fat

Following acclimatization, the initial body weight of the mice was measured after 12 hours of fasting. Subsequently, body weight measurements were recorded once a week during feeding, and the final body weight was obtained by measuring the mice’s weight after 12 hours of fasting just before euthanasia. Weight gain is calculated using the following formula: weight change percentage (%) = (final weight (g) - initial weight (g))/ initial weight (g). After dissection, the intact liver and epididymal fat of the mice were excised and weighed.

#### Measurements of blood glucose and lipid levels

Fasting blood glucose levels in mice were measured weekly using an automated glucometer (ROCHE, Active, Basel, Switzerland). Serum total cholesterol (TC) levels, triglycerides (TG), low-density lipoprotein cholesterol (LDL-C), high-density lipoprotein cholesterol (HDL-C), and glucose (GLU) levels were measured using a microplate reader (Thermo Scientific, MK3, New York, USA) with TC, TG, LDL-C, HDL-C, and GLU kits by the manufacturer’s protocols.

#### Histologic studies

Fresh mouse adipose tissue of epididymis was fixed with fixative for more than 24h and then was dehydrated in alcohol, immersed in paraffin, embedded, and sectioned sequentially. The sections were then dewaxed with xylene, hydrated with gradient concentration ethanol, stained with hematoxylin and eosin, dehydrated with ethanol, transparent with xylene, and sealed with neutral gum. Histopathological changes were observed by microscope (Nikon, Eclipse Ci-L, Tokyo, Japan).

#### Enzyme-linked immunosorbent assay (ELISA)

The serum samples collected were subjected to measurement of interleukin 6 (IL-6), tumor necrosis factor $\alpha$ (TNF-$\alpha$), monocyte chemotactic protein 1 (MCP-1), and glucagon-like peptide 1 (GLP-1) by following the guidelines provided with the ELISA kits.

#### Western blot

Adipose tissue from the epididymis was homogenized and treated with lysate (RIPA: PMSF=100:1) to produce protein extracts. The bicinchoninic acid (BCA) protein kit technique was used to determine the protein concentration in tissue homogenate samples. Sodium dodecyl sulfate-polyacrylamide gel electrophoresis (SDS-PAGE) was used to separate the proteins, which were then transferred to polyvinylidene fluoride (PVDF) membranes. The membranes were incubated overnight with primary and secondary antibodies diluted in TBST. Chemiluminescence imaging was performed by adding developer drops to the PVDF membrane. Protein expression levels of PPAR$\gamma$, SREBP1, UCP1, and TGR5 were analyzed using a gel imager and ImageJ software (Bio-Rad, California, USA).

### Statistical analysis

One-way analysis of variance (ANOVA) was used for the statistical analysis of the data, which were reported as mean ± standard deviation (${\bar{x}}$ ± SD). GraphPad Prism 8.0.1 (Graph Pad Software, Inc., San Diego, CA, USA) was used to create the graphs. Statistical significance was defined as a p-value less than 0.05.

1. Approval: The animal experiments in this study were approved by the Animal Ethics Committee of Guangdong Pharmaceutical University.
2. Accordance: The methods were carried out by the relevant guidelines and regulations.

## Discussion

OA has demonstrated efficacy in the contexts of anti-inflammatory, hypoglycemic, anti-atherosclerotic, and diabetic treatments [43–45]. A systematic review examining OA’s effects on metabolic syndrome-related indicators, such as central adiposity, lipid profiles, blood pressure, hyperglycemia, and biomarkers of oxidative stress, demonstrated its potential in regulating the lipid spectrum and treating insulin resistance and metabolic syndrome [46]. In a previous study, we found that OA can reduce insulin resistance by lowering inflammatory cytokine levels [47]. Moreover, OA has been shown to reduce adipose tissue inflammation by regulating macrophage infiltration and polarization, enhance taste perception, reduce systemic inflammation, promote hepatic adipogenesis, and increase lipid preference [21, 44]. Short-term OA administration to neonatal rats can counteract fructose-induced oxidative stress without impacting long-term health [48]. OA has also been found to regulate redox and PPAR$\gamma$ signaling to reduce PCB-induced obesity and insulin resistance [43]. However, systematic research on its anti-obesity properties is lacking. As a result, we comprehensively investigated the mechanism of OA’s anti-obesity action in this study using network pharmacology, molecular docking, Molecular dynamics simulation and animal trials. We first analyzed and screened the core targets of OA exerting anti-obesity effects using network pharmacology, verified the binding effect of OA to the core targets using molecular docking methods, and further verified the binding stability and binding capacity using molecular dynamics simulations. At the same time, animal experiments were used to verify the core targets and pathways.

The current study is primarily focused on elucidating the underlying mechanism by which OA facilitates weight loss, which is based on the shared target of OA and obesity. A comprehensive search and screening of multiple databases yielded a total of 42 OA-obesity-associated targets, which were subjected to GO enrichment analysis and KEGG pathway enrichment analysis to better comprehend the correlation outcomes. The PPAR signaling pathway was identified as the key pathway based on the results of GO function and KEGG enrichment analysis. The PPAR signaling pathway has been widely recognized as the underlying mechanism of herbs’ lipid-lowering effects [49–51]. The peroxisome proliferator-activated receptors (PPARs) are ligand-activated nuclear hormone receptors [52] that can be activated by fatty acids and their derivatives to regulate the transcription of lipid metabolizing enzymes [53]. PPARs consist of three isoforms, namely PPAR$\alpha$, $\beta$/$\delta$, and PPAR$\gamma$ [54], each of which plays a distinct role in lipid metabolism. PPAR$\alpha$ is primarily present in tissues with high energy demands, such as the liver, kidney, and heart [55]. It mainly regulates the expression of downstream genes and proteins that are associated with lipid metabolism and liver metabolism [56], and is a therapeutic target of fibrates (selective PPAR-agonists) [57]. PPAR$\beta$/$\delta$ is a critical regulator of muscle lipid homeostasis and is involved in systemic lipid regulation by $\beta$/$\delta$ agonists (bezafibrate, telmisartan, etc.) [58]. PPAR$\gamma$ is primarily located in adipose tissue [59] and is a crucial mediator of energy balance and cell differentiation. It can promote adipose differentiation and lipid synthesis, leading to morphological changes and adipocyte enlargement [60, 61]. Furthermore, it is a major regulator of glucose metabolism and a therapeutic target of type 2 diabetes medication thiazolidinediones (TZDs) [53, 61].

Based on the PPI network, we can speculate that PPARG, PPARA, and several other molecules are the primary targets for OA-regulated lipids. PPARG(peroxisome proliferator-activated receptor gamma) and PPARA(peroxisome proliferator-activated receptor Alpha), the aforementioned PPAR$\gamma$ and PPAR$\alpha$, have long been recognized as critical regulators of obesity and are commonly employed in clinical treatment protocols61.MAPK3 (mitogen-activated protein kinase 3), also known as ERK1 (extracellular regulated protein kinases), is a class of intracellular serine/threonine protein kinases that are involved in various cellular processes such as cell proliferation, cell survival, cell growth, cell metabolism, cell migration, and cell differentiation [62]. It plays a role in various diseases such as hepatic lipid metabolism, liver lipid metabolism, cardiac metabolic disorders, etc [63, 64]. Prior research has demonstrated that Leptin, Protocatechuic Acid, and other molecules can combat obesity and atherosclerosis through MAPK3/ERK1 [65, 66]. NR3C1 (Nuclear Receptor Subfamily 3 Group C Member 1), which encodes the glucocorticoid receptor, is involved in the inflammatory response, cell proliferation, and differentiation in target tissues [67]. It has been reported to exert beneficial effects on obesity, impaired glucose metabolism, and dyslipidemia [68–70]. Similarly, PTGS2(prostaglandin-endoperoxide synthase 2) and CYP19A1(cytochrome P450 family 19 subfamily A member 1) are also predicted to be pivotal target for reducing or reversing hyperlipidemia and obesity [68, 71, 72]. Additionally, the CNR1 (cannabinoid receptor 1) has been identified as a promising drug target for the treatment of obesity, with CNR1 knockout in mice resulting in improvements in insulin resistance, ER stress, and lipid accumulation [73]. Conversely, overexpression of HSD11B1 (corticosteroid 11-beta-dehydrogenase isozyme 1), which catalyzes the conversion of the active form of cortisol [74], has been found to result in visceral obesity, insulin-resistant diabetes, and dyslipidemia [75]. AGTR1 (angiotensin II receptor type 1) is a key player in the renin-angiotensin system (RAS) that can increase blood pressure and insulin resistance while also inhibiting lipolysis, maintaining energy homeostasis, and reducing inflammation [76]. Molecular docking and molecular dynamic simulation were used to validate the molecular mechanism of OA intervention in obesity. The molecular docking studies revealed that OA has a high affinity for the key targets identified by network pharmacology. The results of molecular dynamics simulation revealed that OA binding to the core targets was all more stable, indicating that the previously hypothesized core proteins were indeed the critical linkages in OA’s anti-obesity action.

To validate the predicted targets and pathways of OA in treating obesity, we established a high-fat diet-induced obesity mouse model and investigated the effects of OA on inflammatory responses and glucolipid metabolism in mice. We also focused on validating the primary core target, peroxisome proliferator-activated receptor gamma (PPAR$\gamma$), which was identified in our prediction through network pharmacology and molecular docking approaches. Our results demonstrate that OA treatment significantly ameliorated high-fat diet-induced metabolic dysfunction in the treated mice, as evidenced by lowered FBG levels, blood cholesterol levels, tissue damage, and reduced inflammatory response. Our findings are consistent with earlier studies [17, 21, 43]. We also observed that high-fat feeding increased the expression of PPAR$\gamma$ in adipose tissues while decreasing the expression of mitochondrial uncoupling protein 1 (UCP1). Conversely, after OA treatment, the expression of PPAR$\gamma$ decreased, while that of UCP1 increased. UCP1, a downstream target of PPAR$\gamma$, is highly expressed in the mitochondria of brown adipose and beige adipose tissues [77], and is involved in regulating energy expenditure and metabolic dynamic homeostasis through multiple cellular pathways, affecting the production of reactive oxygen species in adipocyte mitochondria [78–80]. The increase in PPAR$\gamma$ and the decrease in UCP1 expression represent an increase in fat synthesis and storage, as well as a decrease in energy consumption in obese mice, which is consistent with the developmental mechanism of obesity. However, the negative effects of these changes were counteracted by the participation of OA. Our findings corroborate previous predictions from network pharmacology and molecular docking on OA’s anti-obesity targets and pathways. Overall, our results suggest that OA can help treat obesity by regulating PPAR$\gamma$ and activating the PPAR signaling pathway.

To gain a better understanding of the effects of OA on lipid accumulation in obese mice, we investigated the regulatory impact of OA on the expression of the proteins SREBP1 and TGR5. SREBP1 (Sterol-regulatory element binding proteins1), a key regulator of fatty acid metabolism, participates in lipid absorption, lipid synthesis, and saturated fatty acid oxidation, and is increased in obese hosts [81, 82]. TGR5, the G-protein-coupled bile acid receptor, controls the metabolism of cholesterol, bile acids, fats, and carbohydrates, as well as insulin and systemic energy expenditure, and has been shown to boost metabolic rate, uptake of oxygen, and decrease obesity and hepatic steatosis in a mouse model of obesity [83–85]. In line with the findings of PPAR$\gamma$/UCP1, obese animals exhibited higher levels of SREBP1 and lower levels of TGR5, whereas OA was able to down-regulate SREBP1 and up-regulate TGR5 expression, suggesting that one possible anti-obesity strategy for OA would involve up-regulating TGR5/UCP1 to encourage fat consumption and down-regulating PPAR$\gamma$/SREBP1 to decrease fat synthesis and alter lipid accumulation. Furthermore, GLP-1 is glucagon-like peptide-1, an enteroglucagon generated from the intestine that increases insulin secretion from pancreatic $\beta$-cells [86] while also decreasing food intake and body weight [87]. It and its receptor have a significant potential for the therapy of glycolipid metabolic disorders. Our study revealed that OA treatment can increase the secretion of TGR5/GLP-1, which supports our previous finding that modulation of TGR5/GLP-1 can improve both inflammatory and glucolipid metabolism disorders. This finding suggests that OA’s ability to combat obesity may involve this mechanism.

In conclusion, this study combines network pharmacology, molecular docking, molecular dynamic simulation, and animal experimental verification for the first time to study the effectiveness and mechanism of action of OA in the treatment of obesity. The findings reveal the potential of OA in the treatment of obesity and provide a novel direction for the development of OA-derived drugs and food, thereby contributing to the discovery and development of natural resources related to obesity. Furthermore, this study explored the effects of medications on obesity through lipid accumulation, providing researchers with new insights for developing obesity drugs. Undeniably, there are some limitations in our study. Network pharmacology, molecular docking, and molecular dynamic simulation are reliant on data and algorithms, and their outcomes may differ from actual results due to database and software limitations. Additionally, due to time and resource limitations, we were unable to experimentally confirm all of the expected targets, or to combine animal and cell research with clinical validation and other methods, preventing us from fully revealing the anti-obesity mechanism of OA. We will conduct additional tests in the future to examine the potential molecular pathways behind the anti-obesity effects of OA in greater detail.

## Conclusion

In conclusion, our study used network pharmacology, molecular docking, MD simulation, and animal trials to uncover putative molecular pathways of OA activity against obesity. The major BP, CC, and MF of OA against obesity were identified using GO enrichment analysis. KEGG/DAVID database enrichment analysis was also used to identify pathways that play key roles in obese OA treatment. The PPI network was used to identify nine main targets, and the potential binding ability and binding stability of OA with each important target were verified using molecular docking technology and MD. PPARG, PPARA, MAPK3, NR3C1, PTGS2, CYP19A1, CNR1, HSD11B1, and AGTR1 were found to play essential roles in the anti-obesity mechanism of OA. The animal experimental validation results also showed that the PPARG and PPAR signaling pathways are critical for the anti-obesity impact of OA on obese mice.

### Supplementary Information

**Additional file 1.**

## Data Availability

The datasets used and/or analysed during the current study are available from the corresponding author on reasonable request.
