# Supplementary figures and images for: Integrating network pharmacology and animal experimental validation to investigate the action mechanism of oleanolic acid in obesity
Source: J Transl Med. 2024 Jan 21;22:86. doi: 10.1186/s12967-023-04840-x (PMC10802007; doi:10.1186/s12967-023-04840-x)

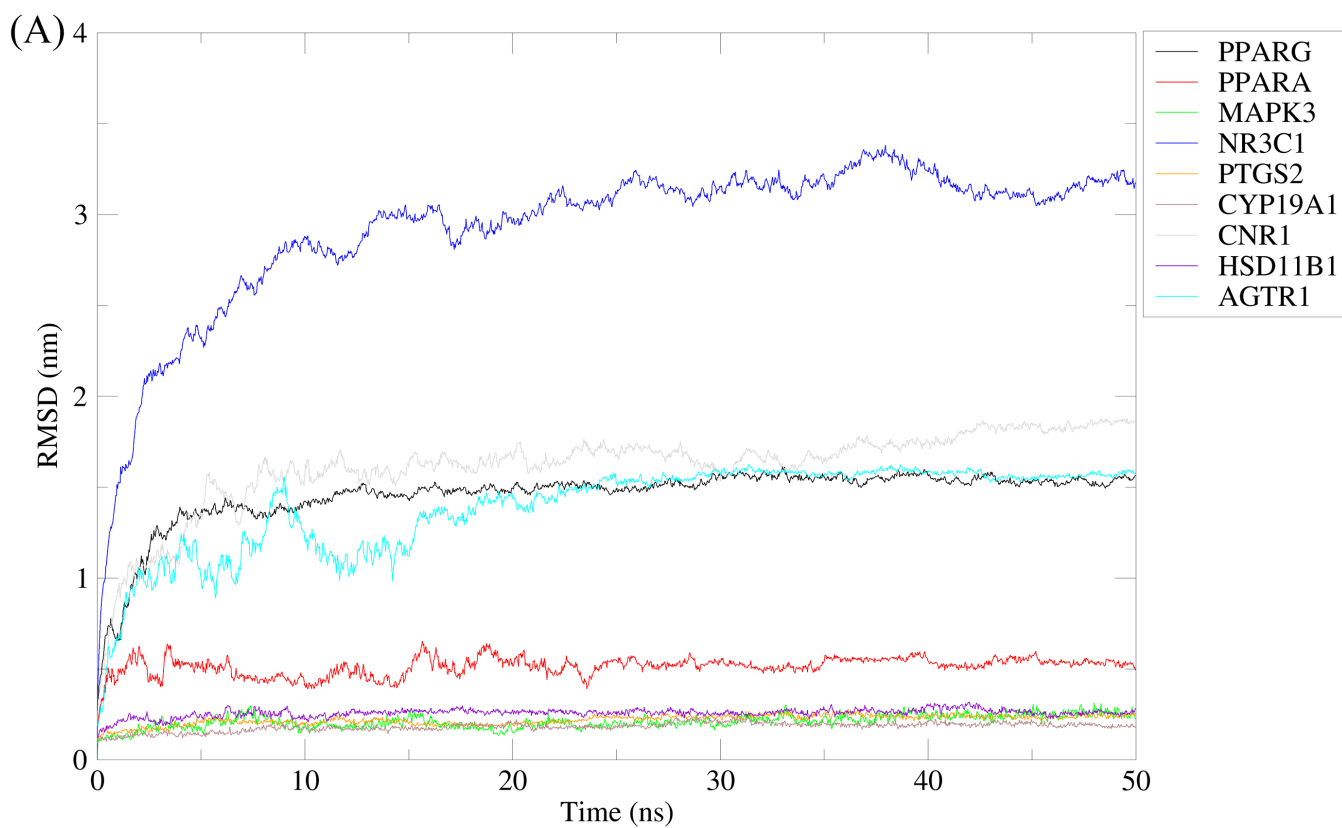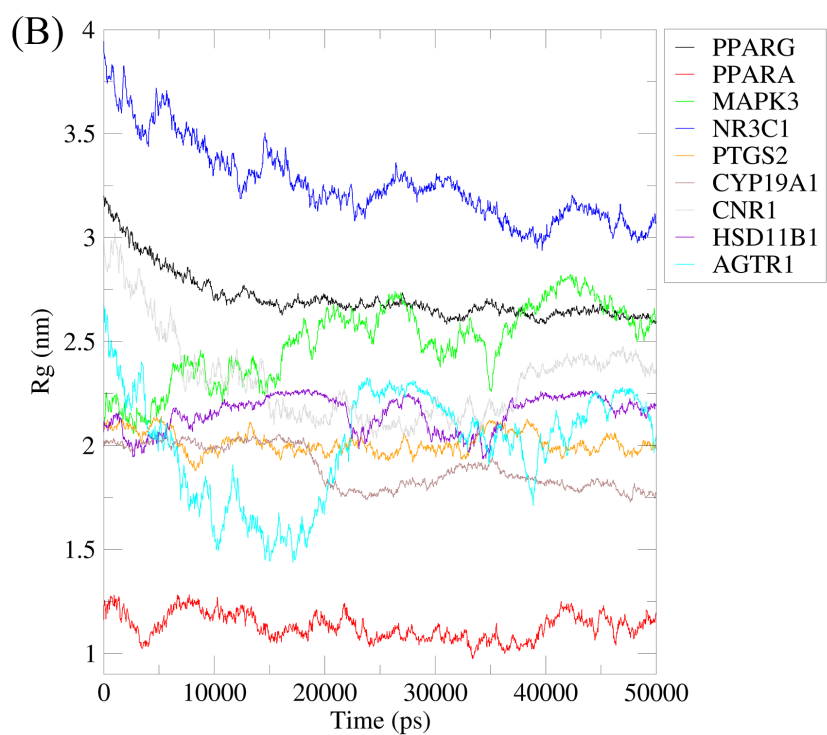

Supplement: Supplementary file 1 — Additional file 1. [file 12967_2023_4840_MOESM1_ESM.pdf]
